# Formation of Blastocysts From Zona Pellucida–Free Oocytes: A Case Report on a Modified Technique in In Vitro Fertilization

**DOI:** 10.1155/crog/5247242

**Published:** 2025-04-25

**Authors:** Minh Tam Le, Trung Van Nguyen, Hong Nhan Thi Dang, Quoc Huy Vu Nguyen

**Affiliations:** ^1^Department of Obstetrics and Gynecology, Hue University of Medicine and Pharmacy, Hue University, Hue, Vietnam; ^2^Center for Reproductive Endocrinology and Infertility, Hue University of Medicine and Pharmacy, Hue University, Hue, Vietnam

**Keywords:** case report, embryo culture, intracytoplasmic sperm injection, zona-free blastocyst, zona-free oocytes

## Abstract

**Objective:** Zona-free oocytes (ZFOs) were rarely present when performing the intracytoplasmic sperm injection (ICSI) technique. There have been some reports showing that embryos from these oocytes still result in pregnancy after treatment. These oocytes are often discarded due to quality concerns and difficulties in manipulating and cultivating. This case report shows how ZFOs are handled in in vitro fertilization (IVF).

**Methods:** This case report concerns IVF with ICSI and blastocyst culture in an infertile woman with polycystic ovary syndrome. A modified ICSI procedure was proposed to fertilize the ZFO without any damage. The primary outcome measurements involve fertilization assessment and blastocyst development.

**Results:** Among 23 retrieved oocytes, 5 of them were zone ZFOs. Three of the five were fertilized and then developed into good-quality blastocysts.

**Conclusions:** By employing appropriate techniques for fertilization and embryo culture, ZFOs are capable of the development and production of good-quality blastocysts.

## 1. Introduction

The zona pellucida (ZP), an extracellular structure of the oocyte, was composed of a translucent glycoprotein matrix. The human ZP was formed from four glycoproteins encoded in genes ZP1, ZP2, ZP3, and ZP4. Functionally, the proteins ZP1, ZP3, and ZP4 bind capacitated sperm and stimulate acrosome reaction, whereas ZP2 binds sperm that has undergone acrosome reaction. Furthermore, during the emergence of fertilization, this structure served to inhibit polyspermy [1]. Physiologically, sperm attached to ZP3 initiates the acrosome reaction and provides the necessary signal to cross the ZP membrane. Sperm and oocyte membrane fusion took place, forming a ZP membrane that became harder and promoted the release of cortical granules into the vitelline region. Alterations to the ZP membrane's structure made it impossible for additional sperm to penetrate and served as the foundation for preventing polyspermy. Ovastacin and protease, two cortical reaction–related enzymes, broke the bonds separating ZP2 glycoproteins, changed the spatial arrangement of ZP3, and thickened and hardened the ZP membrane. The sperm could not identify and attach to the ZP membrane due to structural alterations in ZP2 and ZP3. After fertilization, the sperm that were already adhered to the ZP membrane were prevented from penetrating further by the thickening and hardening of the membrane [1].

An essential function of the ZP membrane is to protect the embryos from mechanical stress before they are implanted. The embryo is exposed to mechanical and chemical stimuli during the migration from the fallopian tube into the uterus. The contractions of the smooth muscles, combined with the oscillations of stimuli, generate fluid flow and create mechanical forces that impact the embryo, potentially disrupting the loose connections between blastomeres during division in the absence of the ZP membrane. The ZP membrane plays a crucial role in providing resistance against these stressors. The ZP functions as a protective barrier, maintaining structural integrity and safeguarding the embryo from compression or injury caused by the repetitive contractions of the smooth muscles in the fallopian tube. The elasticity and rigidity of the embryo maintain its shape and structural integrity against external mechanical stresses while inhibiting premature implantation in the fallopian tube. The ZP regulates the molecular exchange between the embryo and its external environment. It functions as a selective barrier, obstructing detrimental chemicals and immune cells from reaching the embryo while allowing the transfer of vital nutrients and signaling molecules. During assisted reproduction, there was no embryo migration in the fallopian tubes, but manipulation of the embryo load using media droplets and other micromanipulation techniques can impact the embryo. The presence of an intact ZP membrane aids in maintaining the stability of the microenvironment within the embryo, preventing fluctuations in temperature and pH. Additionally, it facilitates improved contact and bonding among blastomeres, leading to compaction [2].

Limited literature is available on what happens when the oocyte is missing the ZP membrane. In the context of in vitro fertilization, zona-free oocytes (ZFOs) are often regarded as abnormal and may be discarded. When employed, the manipulation may be challenging, and there may be issues regarding the poor quality. In 1999, the first case of ZFO was documented by Ding et al. [3]. Nevertheless, this presentation did not report any pregnancy outcomes following embryo transfer. Subsequent reports by Vajta et al., Stanger et al., Shu et al., and Hu et al. confirmed that the ability of embryos created from ZFOs to implant is still ensured [4–7]. Recently, Watson et al. published a report documenting the successful delivery of a healthy girl using the oocyte retrieval process, which involved obtaining entirely ZFOs [2].

Given the rarity and lack of extensive literature on the manipulation and use of ZFOs, we are presenting a case study at our center. The process successfully resulted in the creation of blastocysts from ZFOs, and we anticipate advocating for our manipulation techniques in addressing ZFOs in ART. Appropriate handling of these oocytes will assist the patient in optimizing the probability of obtaining high-quality embryos, increasing the patient's chances of successful therapy.

This case study presented a woman with polycystic ovary syndrome who was unable to conceive. The woman underwent in vitro fertilization using intracytoplasmic sperm injection (ICSI), resulting in the fertilization of three out of five ZFOs. These fertilized oocytes later developed into good-quality blastocysts. The implementation of advancements in embryo manipulation and culture techniques has allowed these ZFOs to offer therapeutic options for patients consistently.

## 2. Case Description

### 2.1. Patient's General Information and History

A couple with primary infertility has been seeking treatment at the Hue Center for Reproductive Endocrinology and Infertility in 2021. The female age was 23 years and diagnosed with polycystic ovary syndrome. Her anti-Mullerian hormone level was measured at 11.0 ng/mL, and she had a normal karyotype of 46XX. The husband was 28 years old and diagnosed with oligoasthenoteratozoospermia and had an aberrant karyotype (46, XY,9qh+).

The patients had no noteworthy familial, surgical, or social history. This IVF cycle was the first treatment cycle for this couple of patients.

### 2.2. Clinical Findings

The woman in our study was diagnosed with polycystic ovary syndrome and underwent ovarian stimulation and oocyte retrieval. The retrieved oocytes exhibited five oocytes with extremely delicate ZP membranes and were classified as empty zona pellucida (EZP). The oocytes were undamaged and possessed a thin ZP, with an average thickness of 10.35 *μ*m. The EZPs were very small, measuring 8.10 *μ*m.

### 2.3. Diagnostic Assessment

The woman's hormonal profiling indicated the follicle-stimulating hormone (FSH) level was 5.66 IU/L, and the level of luteinizing hormone (LH) was 6.92 IU/L. The total antral follicle count (AFC) was 23, with 11 follicles in the right ovary and 12 follicles in the left ovary. The husband underwent a semen analysis, which diagnosed him with oligoasthenoteratozoospermia.

### 2.4. Ovarian Stimulation

The patient underwent in vitro fertilization treatment cycles on November 3, 2021, using a gonadotropin-releasing hormone (GnRH) antagonist protocol. The ovarian stimulation process started on the third day of the menstrual cycle using recombinant FSH (Gonal F, Merck Serono, Germany) at a daily dose of 225 IU. GnRH antagonist (Cetrotide, Merck Serono, Germany) was then administered daily at a dose of 0.1 mg starting on the sixth day of FSH treatment. Following stimulation, ultrasonography monitoring revealed the presence of 32 follicles more prominent than 12 mm. After nine ovarian stimulation days, a dual trigger was employed for oocyte maturation, involving the simultaneous injection of recombinant hCG at 1000 IU and two doses of GnRH agonist (Diphereline, 0.1 mg, France).

### 2.5. Oocyte Preparation

The oocytes were retrieved 36 h after the trigger, using transvaginal ultrasound–guided follicle aspiration with a single-lumen needle (17,107, Vitrolife, Västra Frölunda, Sweden). To minimize any harm to the cumulus–oocyte complexes (COCs) during aspiration, the suction pressure was reduced to 80 mmHg using a craft suction pump (Rocket Medical, England). Following the process of oocyte retrieval, we successfully collected a total of 23 COCs. We rinsed them in 2 mL of G-MOPS PLUS (Vitrolife, Västra Frölunda, Sweden) before immersing them in 2 mL of OVOIL (Vitrolife, Västra Frölunda, Sweden) and finally placed them in an incubator at a temperature of 37°C. After being incubated for 2 h in 1 mL of G-IVF PLUS (Vitrolife, Västra Frölunda, Sweden), which was equilibrated to a temperature of 37°C and a CO_2_ concentration of 5.5%, the COCs were denudated by immersing them in 80 IU of HYASE (Vitrolife, Västra Frölunda, Sweden) for 1 min and then aspirating them using a pipette with a diameter of 140 *μ*m. Following the removal of the outer layers of the cumulus, we recovered 11 intact oocytes in the metaphase II (MII) stage, one oocyte in the metaphase I (MI) stage, one oocyte with a germinal vesicle (GV), six ZFOs, two oocytes with an EZP, and two oocytes undergoing apoptosis. The specimens were placed in 100 *μ*L droplets of G-IVF PLUS and covered with 3 mL of OVOIL for 1 h before ICSI.

### 2.6. Sperm Preparation

Sperm was collected from ejaculation following a period of 3 days of sexual abstinence. The process involves placing sperm in an incubator at a temperature of 37°C for 1 h, resulting in the transformation of semen into a liquid state. The Sil-Select Plus density gradient system (SIP050, Fertipro, Beernem, Belgium) with a 45%–90% density range was used. The semen was subjected to centrifugation at a speed of 300–450×*g* for 10 min. After removing the supernatant, the sperm was rinsed twice with SpermRinse (10101, Vitrolife, Västra Frölunda, Sweden) and centrifuged for an additional 7 min at a force of 300×*g*. After washing, the pellet was resuspended in 0.5-mL SpermRinse, and before ICSI, sperm density and mobility were assessed.

## 3. Therapeutic Intervention

Mature oocytes at the metaphase II stage and ZFOs following removal of the outer layer were utilized for the ICSI procedure with prepared spermatozoa. We have modified the ICSI technique for ZFOs to reduce the risk of damage to these delicate oocytes during the process. The lack of the ZP renders ZFOs more vulnerable to injury from intense suction pressure and results in poor adhesion to the holding needle, complicating the normal ICSI process. To address this issue, we utilized gentle suction pressure to prevent excessive mechanical strain on the oocyte membrane. After the ZFO was delicately aspirated onto the holding needle, sperm was introduced into the cytoplasm utilizing the ICSI needle. Following the sperm injection, the removal of the ICSI needle from the cytoplasm proved challenging due to the insufficient suction power retaining the ZFO. Instead of augmenting the suction pressure on the holding needle, we executed a technical adjustment. The suction pressure on the holding needle was disengaged while the ICSI needle remained within the ZFO cytoplasm. At this juncture, we adjusted the microscope stage to align the oocyte with the equatorial plane of the medium droplet and proceeded to navigate toward the droplet's periphery. The ICSI needle was delicately retracted from the ZFO cytoplasm at the droplet's periphery. This site serves as the interface between the manipulation medium and mineral oil, where surface tension at the medium–oil interface stabilizes the ZFO, aiding in removing the ICSI needle and minimizing the danger of membrane rupture. This approach enhances the recovery and viability of the oocyte, facilitating further development postinjection. These adjustments are essential for guaranteeing the efficiency and safety of the ICSI process for ZFOs.

The injected oocytes were cultured in 100 *μ*L of G-TL (10145, Vitrolife, Västra Frölunda, Sweden) in a Primo Vision culture dish (16606, Vitrolife, Viby, Denmark). The dish was supplemented with 3 mL of OVOIL and placed in an Eppendorf AG Galaxy 170R incubator (Germany) with an atmosphere of 6.0% CO_2_, 5.0% O_2_, and 89.5% N_2_ at 37°C. The analysis of fertilization and cleavage was conducted using a Primo Vision EVO+ starter kit (16621, Vitrolife, Denmark). The embryos were imaged at 10-min intervals in 9 planes until Day 5 following injection.

## 4. Follow-Up and Outcomes

Following the injection of sperm, 8 out of 11 mature oocytes were successfully fertilized, resulting in the formation of 6 blastocysts by Day 5. Three normal fertilizations from ZFOs resulted in the formation of three viable blastocysts. The timelapse observation revealed the delicate nature of cell–cell interaction in culture and did not detect any abnormal embryo cleavage, such as multinucleation (MN—when blastomeres have more than one nucleus), direct cleavage (DC—when a single blastomere divides directly from one to three cells in less than 5 h), or reverse cleavage (RC) in ZFOs. All blastocyst was cryopreserved on the fifth day.

The patient has undergone two embryo transfer cycles. The first time, one good-quality blastocyst was obtained from the patient's mature oocyte, and the patient got pregnant with a *β*-hCG level of 274 mIU/mL. However, she unfortunately experienced a miscarriage at 7 weeks. On the second occasion, two good blastocysts were from intact MII oocytes, and the amount of *β*-hCG was low at 6.2 mIU/mL. Two high-quality blastocysts derived from ZFOs were used during the fourth transfer cycle, yielding a positive *β*-hCG of 500.9 mIU/mL. The clinical pregnancy outcome has been established, and the pregnancy is currently at 13 weeks of gestation.

## 5. Discussion

In practice, ZFOs can be discarded due to challenges in manipulation and uncertainties regarding the quality of the oocytes and embryos they generate. Recorded abnormalities in oocytes, such as the ZFO or a thin ZP membrane, might be associated with mutations [8]. However, the decision to use these embryos from ZFOs for transfer is still being made, taking into account personalized management. The available literature on using ICSI culture and ZFOs for treatment is limited [9]. Nevertheless, new findings on the formation and quality of embryos and the successful transfer of embryos resulting in the birth of healthy babies from ZFOs have opened up possibilities for utilizing these oocytes [2, 7, 9–11].

Concerning factors contributing to the occurrence of ZFO, the first thought is the denudation techniques of COCs. In cases of large oocyte diameter or thin ZP, employing a 140-*μ*m diameter pipette with forceful suction is not advisable since it can potentially harm the oocytes. Larger diameter pipettes, or two needles (21 gauge), separate the oocyte from the COCs. This solution can minimize physical stress on the oocyte [2]. The second significant factor that must be acknowledged is the excessive suction force applied during the oocyte retrieval. Manually extracting follicles has been verified as a potential hazard to delicate COCs. Applying suction pressure and a small diameter needle during follicle aspiration may potentially cause harm to the COCs. The study by Ueno et al. in 2014 demonstrated that a single 21–22-gauge fine needle (Kitazato, Japan) with an aspiration pressure of 300–330 mmHg reported a significant number of 135 cycles presented by ZFOs [12]. The third reason was the cryopreservation and thawing of oocytes. During this procedure, the ZP was disrupted, resulting in the creation of ZFOs [9]. One less common cause of ZFOs is attributed to patient characteristics. According to the study conducted by Zhou et al. in 2019, six couples experiencing primary infertility were found to have a mutation in the ZP gene. Most patients with either ZFOs or a few oocytes with a thin ZP were collected [13]. In the case report by Hu and Trolice [7], only ZFOs were retrieved in two cycles of a patient. The first cycle yielded four oocytes, while the second yielded 11. Recently, another case of Watson et al. reported the acquisition of entire ZFOs from a patient. This case was verified to have a mutation in the gene, namely, heterozygous for Factor V Leiden and Prothrombin 20210 G>A [2].

The patient in our research was diagnosed with polycystic ovary syndrome. Her intact oocytes had a thin ZP membrane measuring, on average, 10.35 *μ*m. In addition, the EZPs we recorded have a tiny size of only 8.10 *μ*m. Upon detecting the first ZFO, we replaced the giant denudation pipette, but the subsequent ZFOs continued manifesting. The cytoplasmic diameter in ZFO (*n* = 5) and intact oocytes (*n* = 11) was 111.32 ± 5.13 and 107.17 ± 2.77, respectively. The ZFOs and oocytes with intact ZP of thin size and EZPs appeared in our case. These ZFOs might have been obtained because these ZP were too thin and easily damaged during egg retrieval or denudation procedures. Furthermore, an intact oocyte was injected within the cultural condition, and the blastomeres escaped from the ZP membrane. Our study observed ZFOs and oocytes with intact, very thin ZP and expanded ZP. The acquisition of these ZFOs may have resulted from the ZP being excessively thin and susceptible to injury during egg extraction or denudation techniques. Furthermore, the blastomeres may escape from the exceedingly thin ZP throughout development. Our case differs from the previously documented case by Hu and Trolice in 2016 and that by Watson et al. in 2021. The ZFOs in their reports were acquired due to defective ZP production, as all recovered oocytes were ZFOs [2, 7].

**Figure 1:**
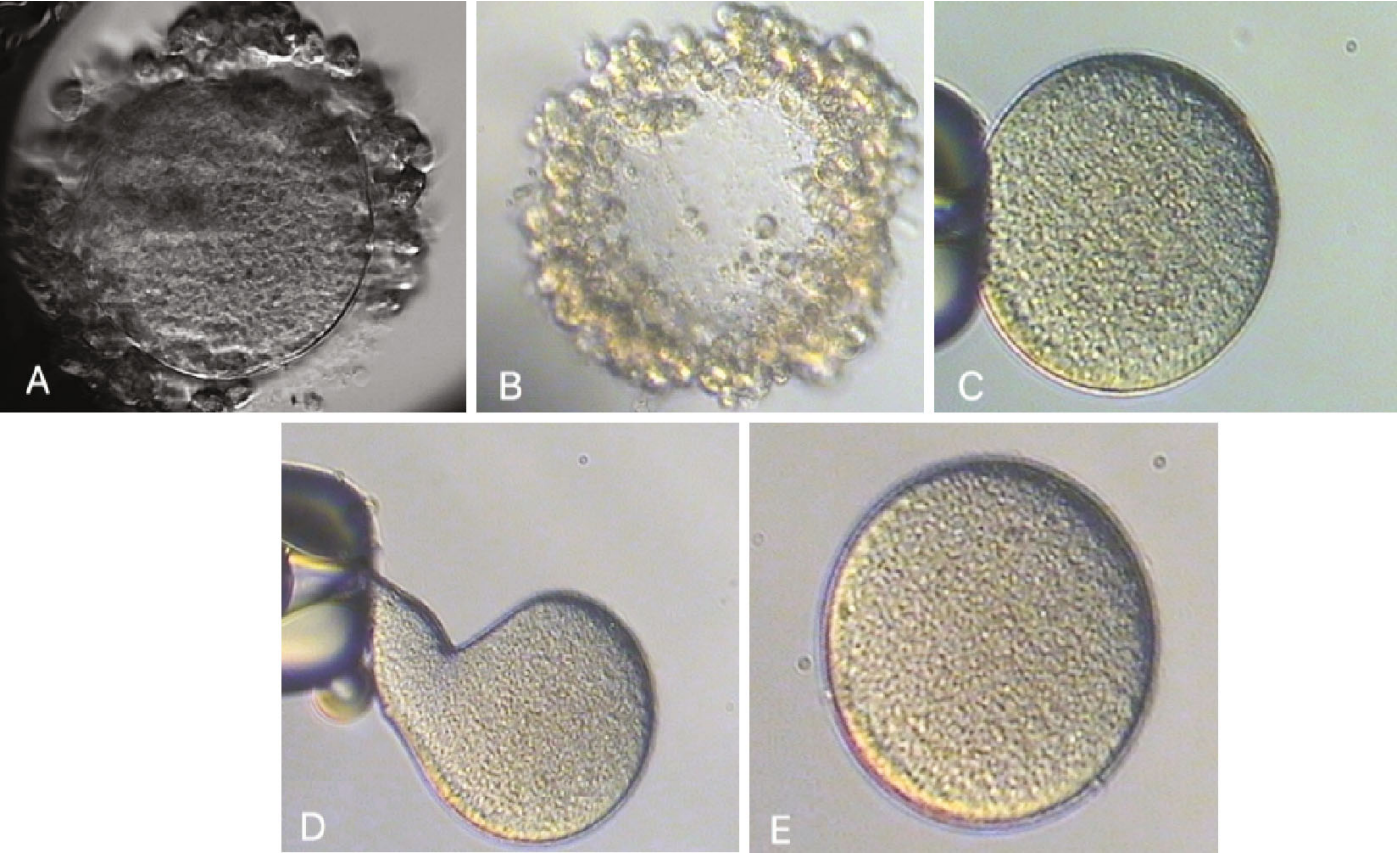
Some typical images of zona-free oocytes and the blastomeres exiting at the weak position of the ZP membrane. (A) Intact oocyte with thin zona pellucida. (B) Empty zona pellucida. (C) Zona-free oocyte. (D) The oocyte was deformed when the ICSI needle was taken out when it was held by holding the needle. (E) After injection, the oocyte morphology did not change when using the ICSI needle to pull back into the interface to remove the ICSI needle.

**Figure 2:**
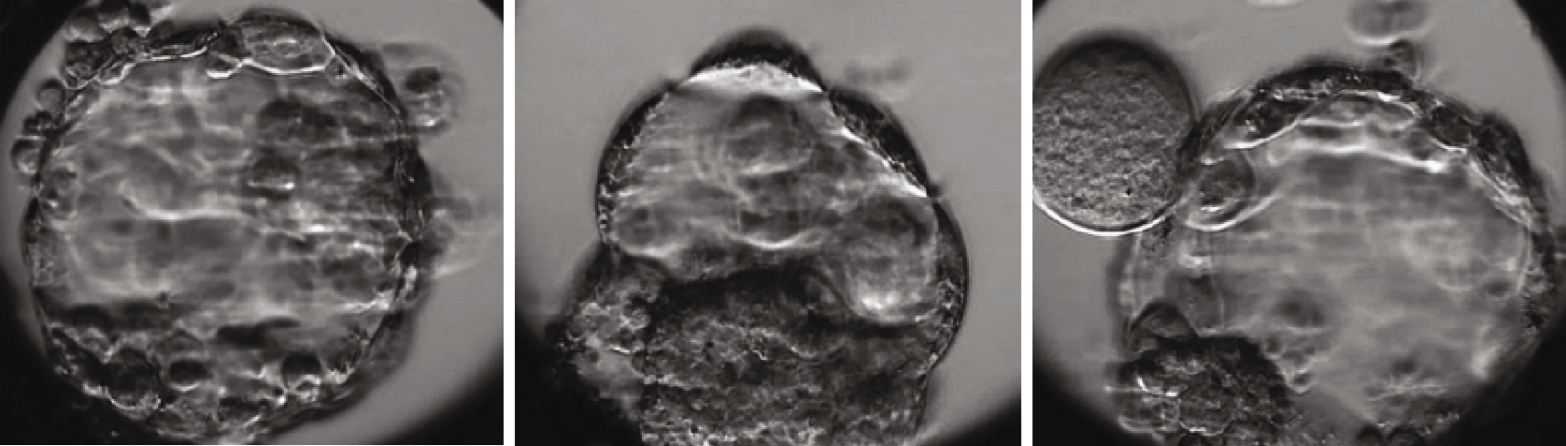
Blastocysts formed from ZFOs.

With the first two ZFOs, we performed the conventional ICSI procedure with the suction force of the holding needle to hold the oocyte during the ICSI needle extracted the cytoplasm. This attraction caused oocyte deformities, resulting in the degeneration of two oocytes following the ICSI. While Hu and Trolice described preserving three layers of corona cells to reduce the suction force of the needle, it is difficult to guarantee the absence of ZFOs and manage the procedural risk [7]. We modified the method by loosely attaching ZFOs to the holding needle. The ICSI needle, after injecting spermatozoa into the cytoplasm, was then brought to the boundary between the medium and the coating oil. This guarantees the isolation of ZFOs from the needle system following ICSI without altering the oocyte morphology (Figure 1). As a result of this technique, the subsequent three ZFOs were unharmed, underwent normal fertilization, and produced good-quality blastocysts (Figure 2).

To minimize the impacts of manipulation and culture of embryos without the ZP membrane after fertilization, we employed a timelapse system for cultivation to monitor the embryo's development. The microwells in the WOW 16 well (Vitrolife, Gothenburg, Sweden) were designed with a smaller diameter to ensure cell–cell contact was maintained during the later cleavage phases, even without the presence of the ZP membrane. It is essential to observe each embryo in a separate state to avoid the fusion of different embryos to create a chimera. Still, blastomeres maintain close contact during the cleavage stage, reorganize during compression, and develop into blastocysts (Movie S1). This culture design guarantees the maintenance of blastomere connectivity throughout development, forming a blastocyst. Further investigation conducted by Song and his colleagues suggests that preserving the interconnection among blastomeres of embryos originating from ZFO oocytes is essential for forming and developing a blastocyst. To mimic the ZP membrane, a well was formed using a double-phase modified sodium hyaluronate gel (MSHG) from Bloomage Biotechnology Corporation Limited, China. This structure facilitates the formation of links between blastomeres during the development process while also enabling the observation of the embryo kinetics [14]. The morphokinetics of embryos obtained from intact oocytes and ZFOs are described in Table 1. In this case, whereas intact oocytes exhibited specific abnormal division characteristics such as DC and multinucleated blastomere, the embryo produced from ZFOs did not display any abnormal expression. The cleavage, compaction, and blastocyst creation time from ZFOs are within the acceptable range, which aligns with the findings of a previous study by Watson et al. in 2021 [2]. Studies conducted on animal models have demonstrated that the development of embryos from the ZFOs did not exhibit any disparities compared to embryos from intact ZP oocytes. They concluded that the ZP membrane is not essential for the in vitro culture of embryos [15, 16].

Considering the previous reports of successful live births from ZFOs, the decision was made to utilize Day 5 embryo cultivation in a timelapse system without any media transfer. This technique prevents superfluous influences throughout the process of culturing, examining embryos, and transferring them using a pipette while minimizing harm to the individual cells when moved without the protective ZP membrane. The records of embryo arrest at the cleavage stage have been documented. The presence of loosely or flattened blastomeres has been found to increase the likelihood of blastomere loss during this stage. Therefore, using blastocyst culture would offer more convenience for the cryopreservation and transfer of embryos [2, 4–7, 9, 11, 12]. Fortunately, all the fertilized ZFOs, in this case, grew into blastocysts on Day 5, as presented in Table 2. Using the single-step medium with specialized timelapse technology to preserve intercellular contact appeared beneficial and resulted in a favorable outcome.

Interestingly, other reports indicated that eliminating the ZP membrane provides certain advantages. In vitro embryo culture induces alterations inside the ZP, including zona hardening and reduced hatching capability. The lack of ZP membrane facilitates embryos' removal of fragmentation and weak blastomeres. Healthy blastomeres readily interact to create a three-dimensional structure, compress, and form a blastocyst [17].

This case report describes a transfer cycle including two high-quality blastocysts derived from ZFOs, which resulted in an ongoing pregnancy. This indicates that utilizing ZFOs retains the potential to develop into blastocysts and offer pregnancy prospects for patients, contingent upon the appropriateness and efficacy of the procedures. This aligns with the findings of Ueno et al. in 2014, which documented nine live births resulting from blastocyst transfer utilizing ZFOs acquired through oocyte retrieval and manipulation [12]. Furthermore, ZFOs resulting from defective ZP production have documented three instances of live babies following treatment [2, 5, 7]. Consequently, while uncommon and perhaps linked to hazards, ZFOs may nonetheless facilitate childbearing possibilities for infertile couples. Our report demonstrated that embryos derived from ZFOs were successfully transferred, leading to a clinical pregnancy for the patient. However, as this study was conducted on a single patient, further research was needed to confirm its applicability. Our observation of embryo development from ZFOs indicated that the cleavage patterns and blastocyst formation rates were comparable to embryos derived from intact oocytes. Nonetheless, the use of embryos from ZFOs should be approached with caution. These oocytes were only considered after all embryos from intact oocytes had been transferred without achieving a successful pregnancy outcome. This report highlights that while embryos from intact oocytes failed to yield a positive pregnancy outcome, transfer embryos from ZFOs resulted in a successful ongoing pregnancy. This finding may serve as preliminary evidence supporting the potential application of ZFO oocytes in future treatments with larger patient populations.

## 6. Conclusions

This report indicated that the fundamental functionalities of ZP membranes can be absent in ZFOs during the ART. Nevertheless, it is still possible to generate good-quality blastocysts by employing appropriate techniques to minimize the impact of manipulation and optimize the culture conditions. No anomalies in blastocyst formation were observed in embryos produced from ZFOs. Therefore, utilizing embryos from ZFOs retains the equivalent potential of embryos from intact oocytes.

## 7. Patient's Perspectives

Receiving ZFOs after oocyte retrieval makes embryologists deeply concerned. The medical team has sought consultation regarding the potential outcomes and unexpected consequences. The blastocyst culture yielded nine viable blastocysts, including the ZFO embryo, which can now be used for embryo transfer. Their embryo transfer cycles using intact oocytes resulted in pregnancy but did not lead to live births. So far, the pregnancy outcome has yielded blastocysts derived from ZFOs. Notwithstanding numerous apprehensions regarding the embryos, we resolved to proceed with their transfer through our specific manipulation and optimized culture conditions. We anticipate the arrival of a healthy baby soon for the infertile couple.

## Figures and Tables

**Table 1:** Features of embryo development in timelapse.

	**ZFO (** **n** = 3**)**	**Intact oocyte (** **n** = 8**)**
tPB2	3.04 ± 1.56	3.00 ± 0.49
tPNa	6.69 ± 1.29	8.53 ± 2.49
tPNf	20.41 ± 2.27	21.08 ± 1.51
t2	22.97 ± 2.19	23.69 ± 2.09
t3	33.14 ± 2.09	32.22 ± 3.83
t4	37.70 ± 3.28	36.33 ± 6.56
t5	45.87 ± 2.94	50.18 ± 11.63
t6	48.32 ± 0.73	49.95 ± 10.23
t7	49.49 ± 1.56	52.86 ± 10.92
t8	51.54 ± 2.69	57.15 ± 13.25
t9	66.76 ± 6.79	68.12 ± 9.49
t9+	67.54 ± 6.72	68.82 ± 8.00
tM	75.93 ± 3.19	81.84 ± 7.11
tSC	93.87 ± 1.93	98.63 ± 8.14
tSB	98.20 ± 4.58	102.29 ± 6.40
tB	104.98 ± 5.66	104.90 ± 6.35
tEB	109.83 ± 4.50	107.79 ± 6.63
MN	—	1
DC	—	1

*Note:* Timing parameters were expressed as hours postinsemination.

Abbreviations: DC, direct cleavage; MN, multinucleation; ZFO, zona-free oocyte.

**Table 2:** Morphological characteristics of blastocysts.

	**ZFO (** **n** = 3**)**	**Intact oocyte (** **n** = 6**)**
Diameter of blastocyst	—	154.67 ± 18.6
Diameter of embryonic structure	141.73 ± 6.86	139.62 ± 27.78
Length of TE	41.33 ± 5.26	33.67 ± 7.95
Width of TE	8.67 ± 1.09	5.47 ± 2.43
S cavity	8024.77 ± 1154.84	11,040.47 ± 5829.72
S ICM	2758.47 ± 1156.19	2955.35 ± 1001.13
S debris	2010.85 ± 1570.7	2070.75 ± 384.31

Abbreviations: ICM, inner cell mass; TE, trophectoderm; ZFO, zona-free oocyte.

## Data Availability

The data supporting this study's findings are available from the corresponding author upon reasonable request.

## Supporting Information

Supporting Information Additional supporting information can be found online in the Supporting Information section. Timelapse recording of the development of embryos from oocytes with and without the zona membrane in a single patient.
